# Recent Advances in Porphyrin-Based Materials for Metal Ions Detection

**DOI:** 10.3390/ijms21165839

**Published:** 2020-08-14

**Authors:** Zhen-Li Qi, Yun-Hui Cheng, Zhou Xu, Mao-Long Chen

**Affiliations:** Hunan Provincial Key Laboratory of Cytochemistry, College of Chemistry and Food Engineering, Changsha University of Science & Technology, Changsha 410114, China; m18373632792@163.com (Z.-L.Q.); chengyh6488@gmail.com (Y.-H.C.); xz_jnu@126.com (Z.X.)

**Keywords:** porphyrin, detection, metal ions, fluorescence, sensor

## Abstract

Porphyrins have planar and conjugated structures, good optical properties, and other special functional properties. Owing to these excellent properties, in recent years, porphyrins and their analogues have emerged as a multifunctional platform for chemical sensors. The rich chemistry of these molecules offers many possibilities for metal ions detection. This review mainly discusses two types of molecular porphyrin and porphyrin composite sensors for metal ions detection, because porphyrins can be functionalized to improve their functional properties, which can introduce more chemical and functional sites. According to the different application materials, the section of porphyrin composite sensors is divided into five sub-categories: (1) porphyrin film, (2) porphyrin metal complex, (3) metal–organic frameworks, (4) graphene materials, and (5) other materials, respectively.

## 1. Introduction

Metal ions are ubiquitous in nature. Metal ions can form biological proteins (e.g., metal proteins and metal enzymes) with biological ligands such as proteins and nucleic acids in the form of ions. They play important biochemical and physiological roles in biological processes. However, excess metal ions (e.g., potassium, calcium, sodium, magnesium, zinc, iron, and other metal elements) and heavy metal ions are harmful to the environment and life. With the rapid development of modern industrialization and urbanization, metal ions, especially heavy metal ions, which are discharged into the ecological environment through domestic sewage and industrial wastewater, waste residues, and waste gas, will cause very serious harm to the environment and life. Therefore, from a biological and environmental perspective, the efficient detection of metal ions remains a serious challenge.

Currently, atomic absorption spectrometry [1], atomic fluorescence spectroscopy [2], inductively coupled plasma-mass spectroscopy (ICP-MS) [3], high-performance liquid chromatography (HPLC), and ion chromatography (IC) are often used for the quantitative detection of metal ions [4,5]. These methods enable the detection and quantification of target analytes with high sensitivity and resolution. However, the instruments are generally costly and require experienced operators, which prevent their wide exploitation in daily life. Therefore, many researchers are working toward the development of convenient and fast metal ions sensors [6,7,8]. In recent years, photochemical sensors (as a high-tech cross-discipline of chemistry, biology, and medicine) exhibit good selectivity, high sensitivity, fast analysis speed, and low cost. They are widely used in the fields of continuous online environmental monitoring and food hygiene inspection [9,10,11,12,13].

Porphyrins are a class of heterocyclic macromolecular compounds formed by the interconnection of the α-carbon atoms of four pyrrole-like subunits through a methine bridge. The porphyrin ring has 26 π electrons and is a highly conjugated system. It has strong light absorption and emission characteristics, especially after complexing with metal ions. Since porphyrins contain a large conjugated system and possess chromophores, they are sensitive to small changes that can be detected via ultraviolet, fluorescence, and other spectroscopic techniques. The characteristic peaks of porphyrins in the ultraviolet-visible spectrum are a strong absorption peak in the range of 400–420 nm (Soret band) and four weak absorption peaks in the range of 500–700 nm (Q band). These peaks are related to the π/π* electron transfer of porphyrin unsaturated ligands. Various substituents can be attached to the outer ring of porphyrin, which can be utilized to develop several porphyrin-based sensors. The central metal can be changed [14], and other molecules or groups can be grafted to the porphyrin ring [15,16]. In general, porphyrins are not only easy to modify, but also have strong stability and perform various biological functions [17,18,19]. Thus, porphyrin-based sensors can be used for biomolecule detection [20], anion detection [21,22], and cation detection [23,24,25]. The porphyrin molecule can be used not only as a sensor to detect analytes directly, but also as a dopant of other materials to form a composite sensor to make a more stable and productive sensor element. Porphyrin-based composite sensors can provide fast, on-site, and real-time analysis. The purpose and significance of this review are to summarize the recent applications of porphyrin-based sensors developed by porphyrins in the detection of metal ions and provide outlooks for future research.

Porphyrin-based materials have been reviewed as chemical sensors [26], molecular probes [27], and metalloporphyrins for environmental and energy applications [28], and Ding et al. [29] had reviewed the application of porphyrin analogues in the detection of cations and anions. This review only focuses on the recent applications of porphyrins and porphyrin composite materials in metal ions detection. Summarized porphyrins and porphyrin-based materials for metal ions detection are provided in Table 1. 

## 2. Porphyrin-Based Fluorescent Molecular Probes for Metal Ions Detection

### 2.1. Porphyrin Molecule by Functional Modification

Porphyrin has a large planar conjugated system, which gives it good optical properties. Thus, the porphyrin molecule is a good class of metal ions probes. However, most porphyrins are water-insoluble, which limits their application in metal ions analysis and detection. At present, cationic water-soluble porphyrins can be formed by the protonation of pyridyl groups and amino groups in porphyrins; or, sulfonate groups can be introduced through sulfonation, and anionic water-soluble porphyrins can also be obtained by the hydrolysis of carboxylate groups. In short, various types of functional groups can be introduced into the porphyrin system through simple reactions such as acylation, substitution, or coordination to meet the functional structural design requirements of the porphyrins.

**Figure 1:**
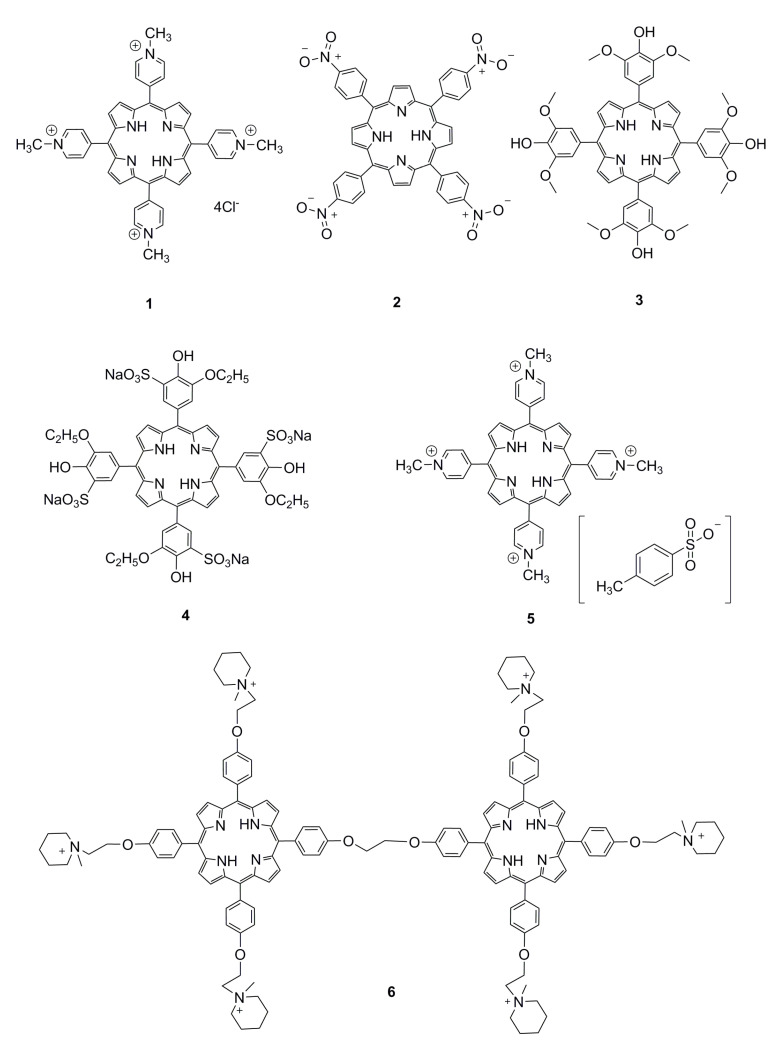
Ionic porphyrins. Reproduced from ref [32,34,39,43,81,82].

Therefore, it is possible to improve the water solubility and ion selectivity of porphyrins by functional modification. Zamadar et al. [43] have used cationic 5,10,15,20-tetrakis (*N*-methyl-4-pyridyl) porphyrin tetrachloride (**1**) (Figure 1) as an optical sensor that could simultaneously detect Hg^2+^, Pb^2+^, Cu^2+^, and Cd^2+^ using UV-vis spectroscopy. Cu^2+^ is mainly attached to the center of the porphyrin ring, while Pb^2+^, Hg^2+^, and Cd^2+^ cannot enter the porphyrin center due to their large ionic radii. This water-soluble cationic porphyrin solves the problem of porphyrin water solubility; however, it does not have good metal ions selectivity. Khani et al. [81] have synthesized and used tetrakis (4-nitrophenyl) porphyrin (**2**) (Figure 1) as a fluorescent probe to detect Cd^2+^ in an ethanol solution. The change in the fluorescence spectrum of **2** is due to the interactions of the four nitro groups of the porphyrin with the Cd^2+^. Namitha et al. [32] have synthesized a water-soluble 5,10,15,20-tetrakis (4-hydroxy-3,5 dimethoxyphenyl) porphyrin (**3**) from 5,10,15,20-tetrakis (3,4,5-trimethoxyphenyl) porphyrin (TMPP) by partial demethylation. The interaction of Cd^2+^ and **3** dynamically quenches the fluorescence of **3** through the electron transfer mechanism to realize the detection of Cd^2+^. Feng et al. [39] have synthesized sulfonate porphyrin and interacted with the center of the porphyrin ring through Mo^6+^, Mo^6+^ induced H aggregation, and the fluorescence spectrum of **4** changed. Sallam et al. [82] have used 5,10,15,20-tetrakis (*N*-methyl-4-pyridyl) porphyrin toluene sulfonate (**5**) to detect metal ions in different solutions. This cationic porphyrin has different absorption in the ultraviolet visible region for Hg^2+^, Pb^2+^, Cd^2+^, and Cu^2+^. Liu et al. [34] have used 5,10,15,20-tetrakis (4-hydroxyphenyl) porphyrin (THPP) as raw materials to synthesize Bis-TMPipEOPP (**6**) by four steps. By modifying the peripheral substituents, they used piperidinyl to replace the phenolic hydroxyl group and finally methylated the peripheral substituents to obtain water-soluble cationic porphyrins, and with specifically the detection of Cu^2+^ ions by fluorescence quenching. These water-soluble porphyrins are usually soluble in hydrophilic solvents such as water, methanol, ethanol, acetone, and acetonitrile. Thanks to pyridyl, sulfonic acid, amino, carboxyl, and other large polar groups have good water solubility. 

**Figure 2:**
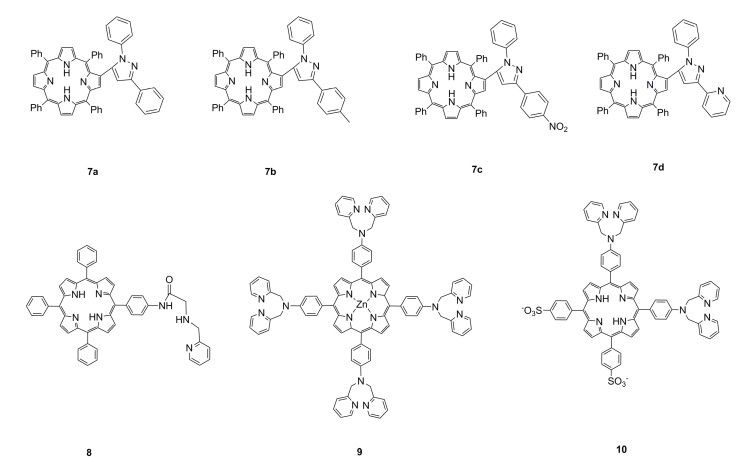
Structural formula of porphyrins modified with pyrrole, pyrazole, and pyridine. Reproduced from ref [31,33,84,85].

Pyridine, pyrrole, and pyrazole rings that contain NH groups can be used as binding sites for metal ions. Therefore, these molecules are often introduced when functionalizing porphyrins. Thus, sensors for detecting metal ions have been developed. The structural formulas of these functionalized porphyrins are shown in Figure 2. Tiwari and Nath [83] have synthesized pyrrolo [1,2-a] pyrazinoporphyrin zinc, and a significant red shift occurred in the Soret band after adding a mercury chloride solution to its acetonitrile solution. In addition, it can be visually observed that the acetonitrile solution changes color from yellow-brown to green and has a considerable specific effect on Hg^2+^. Moura et al. [84] have synthesized a series of pyrazole–porphyrin conjugates. The structural formula (**7a**–**7d**) is shown in Figure 2. The conjugate used a tetrapyrrole core and pyrazole moieties as signal fluorophores and metal ions receptors. After interacting with Zn^2+^, there is a considerable change in the fluorescence peak ratio; upon the pyrazole–porphyrin ligand binding with Cu^2+^, their fluorescence is quenched. Lv et al. [85] used a picolylamine group to functionalize porphyrin as a sensor (**8**) (Figure 2) for detecting Cd^2+^. The three nitrogen atoms in the picolylamine group generated a binding pocket for Cd^2+^. The sensor exhibited selectivity and sensitivity toward Cd^2+^ in a moderate pH range and showed potential for both naked-eye (the color can change from pink to yellow-green) and ratiometric fluorescent detection. Weng et al. [33] have used zinc porphyrin as a fluorophore and 2,2’-dipyridylamine (DPA) as a selected binding site for metal ions and developed a copper ion sensor 5,10,15,20-tetrakis [(*p*-*N*,*N*-bis(2-pyridyl)amino)phenyl] zinc porphyrin (**9**) (Figure 2). It displayed selectivity and anti-disturbance for Cu^2+^ among the metal ions examined (Na^+^, Mg^2+^, Cr^3+^, Mn^2+^, Fe^2+^, Co^2+^, Ni^2+^, Cu^2+^, Ag^+^, Zn^2+^, Cd^2+^, Hg^2+^, and Fe^3+^) in a methanol solution, and its fluorescence can be revived by the addition of an ethylenediaminetetraacetic acid (EDTA) disodium solution. Huang et al. [31] have modified 5,10-bis(4-aminophenyl)-15,20-diphenylporphyrin by DPA groups; the probe (**10**) (Figure 2) showed selectivity toward Cd^2+^ ions in aqueous solutions at physiological pH with a relatively low limit of detection (LOD). The interaction of porphyrin derivative with Cd^2+^ was completed in less than 5 min. Moreover, the optical response of porphyrin derivative to Cd^2+^ was determined to be completely reversible by adding an appropriate amount of EDTA. 

**Figure 3:**
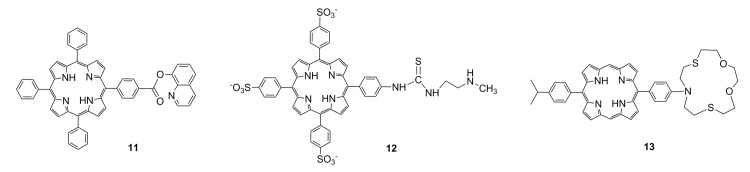
Structural formula of porphyrins modified with sulfur and oxygen atoms. Reproduced from ref [35,36,42].

In addition to nitrogen-containing five- or six-membered rings, metal ions easily combine with sulfur and oxygen atoms. The structural formulas of these functionalized porphyrins are shown in Figure 3. Han et al. [35] have synthesized a compound quinolin-8-ol *p*-[10’,15’,20’-triphenyl-5’-porphyrinyl] benzoate (**11**) (Figure 3) as a ratiometric fluorescent sensor for Hg^2+^ in aqueous ethanol. The Hg^2+^ ion is partially combined with quinoline, which quenches the fluorescence of porphyrin at 646 nm, and a new fluorescence enhancement is induced at 603 nm. Santos et al. [86] have synthesized a porphyrin coumarin conjugate. The inclusion of coumarin nucleus in the porphyrin skeleton enhances fluorescence emission and provides better solubility in aqueous media. Coumarin transfers energy to porphyrins and extends the fluorescence lifetime. This compound can detect Hg^2+^ in an aqueous ethanol solution. Lv et al. [36] have synthesized a new thiourea derivative (**12**) (Figure 3) bearing a water-soluble porphyrin fluorophore; the sensor was used as a chemodosimeter for Hg^2+^ in an aqueous solution with an LOD on the ppb level. Upon excitation at 365 nm under a UV lamp, the deep red fluorescence of thiourea derivative became orange only in the case of Hg^2+^. In addition, it exhibited membrane permeability and could be used for the confocal fluorescence imaging of intracellular Hg^2+^ in living cells. In 2013, Chen [42] designed and prepared a novel azacrown [N,S,O]-substituted metal-free porphyrin compound (**13**) (Figure 3) for the simultaneous detection of Ag^+^, Pb^2+^ (*I*460/*I*415, *I*460/*I*551, and *I*460/*I*507), and Cu^2+^ (*I*531/*I*507, *I*531/*I*551), in particular for Cu^2+^ with a dual-mode multisignal detecting potential. The detection limit of this sensor for Cu^2+^ is 2.6 × 10^−13^ M. (*I* is expressed as the fluorescence intensity at a certain wavelength, which is mostly used in proportional fluorescence detection.)

### 2.2. Detecting Mechanism of Fluorescent Porphyrin Molecular Probes

In terms of fluorescence detection, common mechanisms include photo-induced electron transfer (PET), fluorescence resonance energy transfer (FRET), intra-molecular charge transfer (ICT), and aggregation-induced luminescence (AIE). In addition, there is an excited intra-molecular proton transfer (ESIPT). From the literature, the most commonly involved detection principle of porphyrin-based fluorescent sensor is FRET, followed by PET, which we will introduce in detail below.

**Figure 4:**
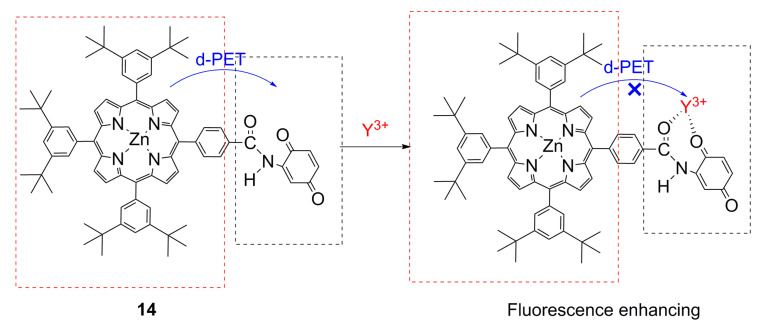
Schematic of of zinc porphyrin–quinone-linked dyad for detection of Y^3+^. Reproduced from ref. [44].

In PET fluorescent molecular probes, there is a photo-induced electron transfer between the fluorophore and the acceptor unit, which has a very strong quenching effect on fluorescence, usually featuring an electron transfer from the donor to the excited state fluorophore. Therefore, without binding to the guest, the probe molecule does not emit fluorescence, or the fluorescence is weak. Once the receptor is combined with the guest, the photo-induced electron transfer is inhibited or even completely blocked, and the fluorophore emits fluorescence [87,88]. Many metalloporphyrins can act as electron donors. Okamoto and Fukuzumi [44] have synthesized a zinc porphyrin–quinone-linked dyad. This structural formula is shown in Figure 4. When compound (**14**) (Figure 4) was combined with Y^3+^, Y^3+^ partially bonded to quinone, which hindered light-induced electron transfer and enhanced the fluorescence of the probe. Singh Virk et al. [37] have synthesized and used the strong binding effect of sulfur atoms and Hg^2+^ in tetrathia porphyrin, which reduced the intensity of the corresponding electronic transitions, reduced the intensity of the ultraviolet absorption band of the compound, and enabled the colorimetric detection of Hg^2+^ with an LOD of 0.04 ppb. 

**Figure 5:**
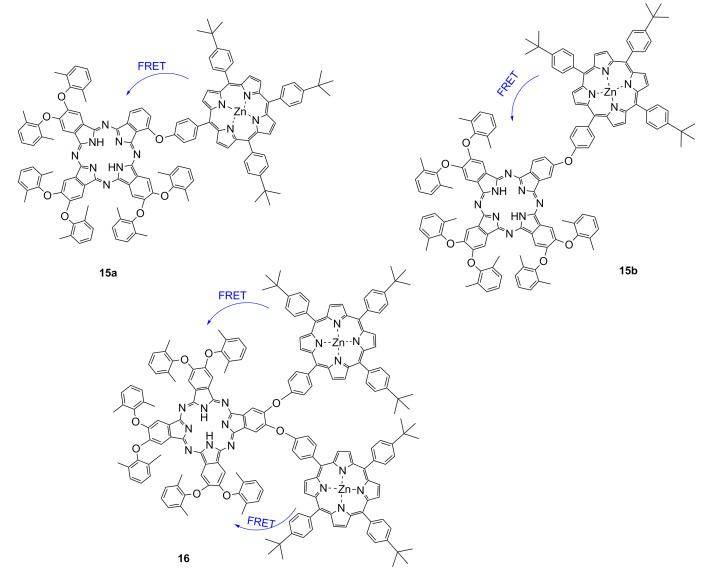
Schematic of phthalocyanine−porphyrin heterodyads for the detection of Pb^2+^ ion by fluorescence resonance energy transfer (FRET). Reproduced from ref [38,40,41].

**Figure 6:**
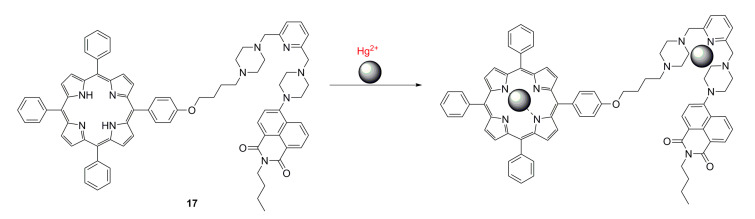
Schematic of naphthalimide–porphyrin hybrid for the ratiometric detection of Hg^2+^ ion. Reproduced from ref. [38].

**Figure 7:**
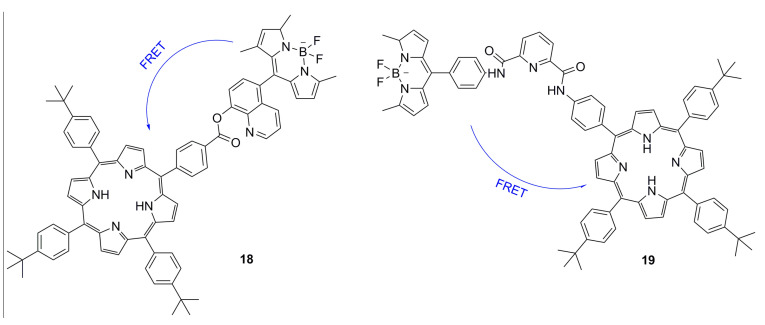
Schematic diagram of BODIPY–porphyrin dyad for the detection of metal ions by FRET. Reproduced from ref [30,91].

**Figure 8:**
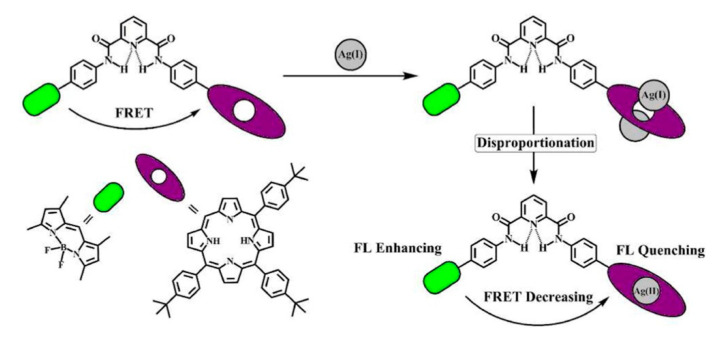
Sensing mechanism of the BODIPY–porphyrin dyad toward Ag^+^. Reprinted with permission from ref [30]. Copyright 2014 American Chemical Society.

Fluorescence resonance energy transfer occurs in two different fluorescent groups. If the emission spectrum of one fluorescence group (donor) overlaps the absorption spectrum of another fluorescence group (acceptor), when the distance is appropriate, the phenomenon of fluorescence energy transfer from donor to acceptor can be observed. If the acceptor is also a type of fluorescence emitter, the receptor will exhibit fluorescence, and a double emission wavelength can be observed [89,90]. Therefore, a donor and receptor can be designed as a molecule by covalent binding. Owing to the optical properties of porphyrins, a series of fluorescent proportional sensors can be developed on the basis of the principle of resonance transfer of fluorescence energy. The structure of these fluorescent sensors is shown in Figure 5. Zhang et al. [40] have designed FRET-based phthalocyanine–porphyrin (Zn) heterodyads for the ratiometric fluorescent detection of Pb^2+^. Both H_2_Pc-α-ZnPor (**15a**) (Figure 5) and H_2_Pc-β-ZnPor **(15b**) (Figure 5) feature a highly efficient FRET process that could be suppressed by the selective binding of Pb^2+^ in the phthalocyanine core, which leads to the enhancement of donor (ZnPor) emission and diminishing of acceptor (phthalocyanine) emission. The LOD for detecting Pb^2+^ can be decreased to sub-parts per billion. Qi et al. [41] have used the same principle to design the phthalocyanine–porphyrin hetero-triad H_2_Pc-β-(ZnPor)_2_ (**16)** for Pb^2+^ ions detection. Li et al. [38] have synthesized a naphthalimide–porphyrin hybrid (**17**) (Figure 6) for the ratiometric detection of Hg^2+^ in an aqueous solution and living cells. This sensor has two units. The first one is the 2,6-bis(aminomethyl) pyridine unit, which adopts a semirigid V-shaped conformation that may be able to selectively bind with Hg^2+^. The other one is the porphyrin unit, whose tetrapyrrolic center can coordinate with Hg^2+^. The schematic diagram of the naphthalimide–porphyrin hybrid for the ratiometric detection of Hg^2+^ is shown in Figure 6. This hybrid probe achieved well-resolved emission spectra with a 125-nm difference between two emission peaks (*I*525/*I*650), which benefited from the observation of fluorescence signal change at two different wavelengths with high resolution. It is worth noting that boron–dipyrromethene (BODIPY) has been widely employed as a good signal moiety of a fluorescence sensor owing to its advantageous photophysical characteristics. Chen et al. [91] have designed a flexible 8-hydroxyquinoline benzoate-linked BODIPY–porphyrin dyad and determined that an 8-hydroxyquinoline benzoate (8-HQ-B)-linked BODIPY-(8-HQ-B)-porphyrin dyad (**18**) (Figure 7) exhibited versatile selectivity for Hg^2+^ and Fe^2+^ owing to the opposite effect on the intra-molecular energy transfer upon binding with these two cations. Similarly, BODIPY fluorophore serves as a FRET donor, and porphyrin fluorophore serves as an acceptor. Zhu et al. [30] have used a BODIPY–porphyrin dyad (**19**) (Figure 7) for the ratiometric fluorescent detection of Ag^+^. It showed a highly sensitive response toward Ag^+^ in the range of 1.0 × 10^−6^–2.0 × 10^−5^ M and with an LOD of 2.0 × 10^−7^ M. The mechanism of the BODIPY–porphyrin dyad for Ag^+^ detection is shown in Figure 8. There are three reasons for the change in the spectrum. The first reason is the quenching of the emission of the porphyrin acceptor. The second reason is the movement of the absorption of porphyrin, which leads to a reduction in the spectral overlap between the emission of the BODIPY donor and the absorption of the porphyrin acceptor, which diminishes the FRET efficiency. The third reason is that it may enhance the emission of the BODIPY donor.

## 3. Construction of Porphyrin-Based Materials for Metal Ions Detection

As a fluorescent organic molecule, porphyrins and metal porphyrins can be functionalized by introducing functional groups to modify the porphyrin ring or use electron energy transfer to connect other dye molecules, so they can be used as a fluorescent probe for detecting metal ions. At the same time, porphyrins are organic ligands that are sensitive to metal ions. They can also participate in the design of other materials, such as composite membranes, metal complexes, metal–organic framework materials, graphene, and other materials.

### 3.1. Porphyrin Film

The porphyrin film can be used as a membrane electrode in an electrochemical method [92,93,94,95], and sol–gel films can be made as photochemical sensors by a sol–gel method [50,96]. Among them, the more common films are polymer films formed by combining an organic or inorganic compound with a high-molecular polymer. The polymer films can be divided into hydrophobic polymer films (polystyrene, polyvinyl chloride, polyethylene, silicone rubber) and hydrophilic polymer membranes (cellulose, agar, polyacrylate, polyacrylamide). As a result of their good mechanical properties and stability, these polymer membranes are often used in gas, ion, and biosensing [97,98,99].

**Figure 9:**
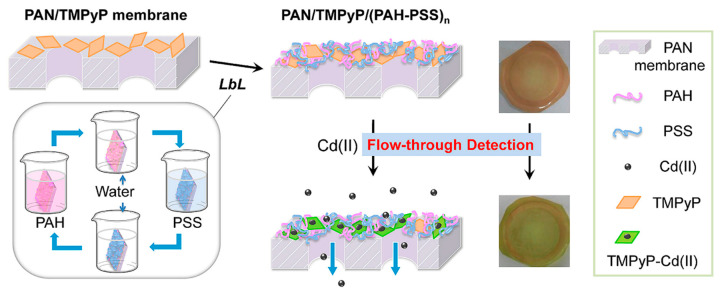
Schematic model of optical sensor membrane fabrication and flow-through detection of Cd^2+^ ions. Reprinted with permission from ref [45]. Copyright 2019 Elsevier Ltd. All rights reserved.

**Figure 10:**
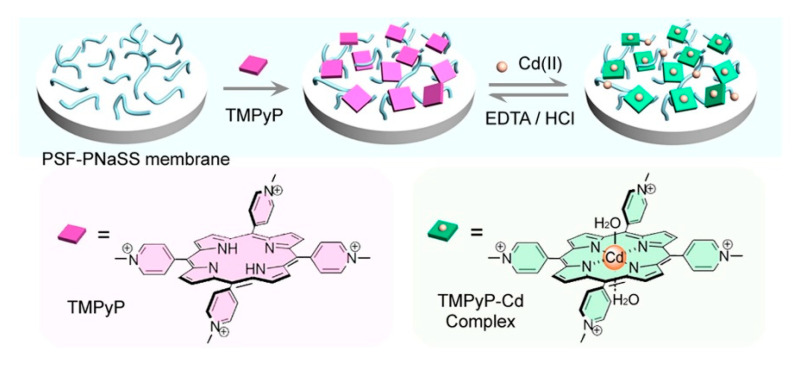
Schematic model of the polyanionic poly (sodium 4-styrenesulfonate)-grafted polysulfone (PSF-PNaSS)/5,10,15,20-tetrakis (4-*N*-methylpyridinyl) porphyrin *p*-toluenesulfonate (TMPyP) membrane fabrication and the sensitive response to Cd^2+^ ions. Reprinted with permission from ref [46]. Copyright 2015 Elsevier B.V. All rights reserved.

In recent years, the doping of organic dye molecules within polymer matrices has gained interest. These matrices are easily prepared and provide immobilization. In addition, through this immobilization, the ground and excited state properties of single molecules can be understood. In favorable cases, the immobilization of fluorescent molecules in the solid matrix may reduce intra-molecular motions and rearrangements, which leads to enhanced photostability and fluorescence capability. Porphyrins can be used as highly sensitive indicators [100]. In 1990, R. Czolk et al. [101] have begun developing fiber optic sensors for the detection of heavy metal ions using porphyrins. They used 5,10,15,20-tetrakis-(*p*-sulfonatophenyl) porphyrin (TPPS) to develop a cadmium-sensitive optical sensor [47]. The fiber optic sensor could sensitively and selectively detect Cd^2+^ in an aqueous solution. In 2002, Zhang et al. [49] have synthesized 5-*p*-[[4-(10’,-15’,20’-triphenyl-5’-porphinato)phenyloxyl]-1-butyloxyl]-phenyl-10,15,20-triphenylporphine (DTPP) and developed an Hg^2+^-sensitive optical fiber chemical sensor in which Hg^2+^ could strongly quench the fluorescence of a DTPP-containing membrane. Porphyrins are deposited on the surface of silanized glass to form porphyrin films [50], self-assembled films [45,51,102], and porous polyfluorene films [46,48]. They are applied to the optical sensor films for heavy metal ions such as Hg^2+^ and Cd^2+^. A schematic model of optical sensor membrane fabrication and flow-through detection of Cd^2+^ is shown in Figure 9. Zhao et al. [46] have grafted polyanionic poly (sodium 4-styrenesulfonate) (PNaSS) onto a chloromethylated polysulfone (CMPSF) microporous membrane via surface-induced atom transfer radical polymerization (ATRP). 5,10,15,20-tetrakis (4-*N*-methylpyridinyl) porphyrin *p*-toluenesulfonate (TMPyP) was fixed on a PNaSS-grafted polysulfone (PSF-PNaSS) membrane by electrostatic interaction. Using this film, Cd^2+^ can be detected through color depth and optical changes. The schematic model of PSF-PNaSS/TMPyP film manufacturing and the principle of Cd^2+^ induction are shown in Figure 10. The obtained porphyrin-functionalized membrane enables enhanced sorption and the sensitive detection of Cd^2+^.

### 3.2. Porphyrin Metal Complex

**Figure 11:**
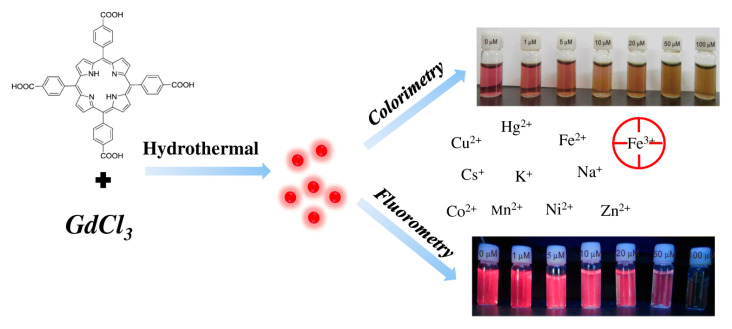
Schematic illustration of Gd−TCPP (5,10,15,20-tetrakis (4 carboxyphenyl) porphyrin) nanosized coordination polymer for Fe^3+^ detection in the colorimetric and fluorometric modes. Reprinted with permission from ref [53]. Copyright 2019 Springer link.

Coordination polymer (CP) is a hybrid material, which is formed by organic ligands and metal ions, and it has a certain stability. Lanthanide metal ions or fluorescent ligands are often selected to synthesize fluorescent coordination polymers to detect gases, ions, and toxic compounds. In 2016, Ramin Boroujerdi [52] used tetraphenylporphyrin (TPP) as ligand to synthesize a Ce–TPP complex by a one-step method. Ce_2_(TPP)_3_ was synthesized to detect Cu^2+^ and Hg^2+^ in aqueous media by changing the color, which also can be used as a selective mercury naked-eye sensor. Porphyrins can be used as organic ligands to coordinate with Gd^3+^ to form coordination compounds to detect trivalent Fe^3+^. Chen et al. [53] have prepared a Gd–TCPP (5,10,15,20-tetrakis (4 carboxyphenyl) porphyrin) coordination polymer in a one-step hydrothermal treatment procedure, which is shown in Figure 11. The apparent color changes of solution (from red to brown) were observed with the addition of Fe^3+^ as low as 5 μM under room light. The assay was applied to the determination of Fe^3+^ in fetal bovine serum samples, and the detection time was less than 3 min.

### 3.3. Metal–Organic Frameworks

Metal–organic frameworks (MOFs) are porous crystalline materials with three-dimensional framework structures formed by the self-assembly of metal ions or metal clusters with organic ligands. MOF materials have the advantages of large specific surface area, many adsorbable sites, strong designability, and functional modification [103,104,105]. They are used in the fields of chemical sensing [106], electrochemical detection [107], catalysis [108], gas adsorption and separation [109], and drug loading [110]. Continuous progress has been made and has received widespread attention in recent decades. MOF materials with excellent luminescence performance and stable structure can be used as fluorescent probes. Groups that can specifically recognize metal ions can be introduced through the selection of fluorescent ligands or post-synthesis modifications, which achieves the highly selective detection of metal ions by quenching or enhancing fluorescence intensity. Therefore, in recent years, MOFs have been rapidly developed in the field of ion fluorescence detection.

**Figure 12:**
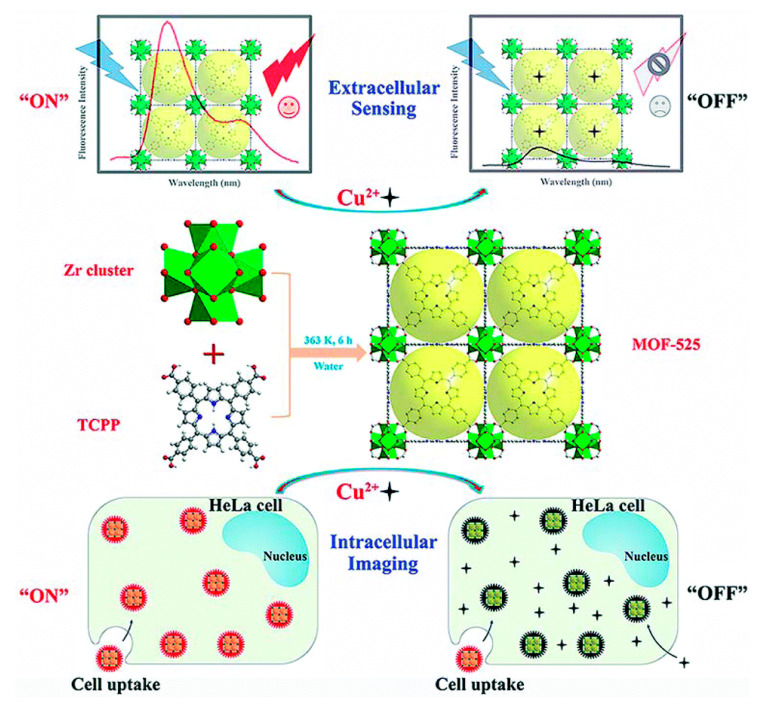
Schematic illustration of the preparation of MOF-525 NPs and the “signal-off” fluorescence sensing for Cu^2+^. Reprinted with permission from ref [58]. Copyright The Royal Society of Chemistry 2019.

**Figure 13:**
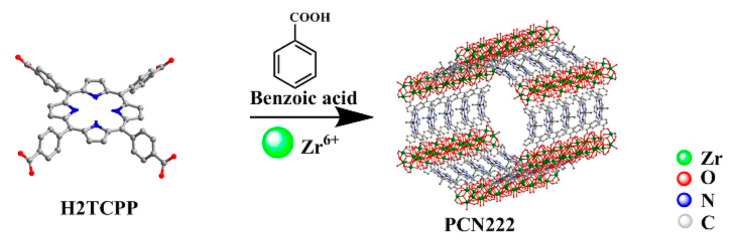
Schematic diagram of PCN222 preparation process. Reprinted with permission from ref [59]. Copyright 2019 American Chemical Society.

**Figure 14:**
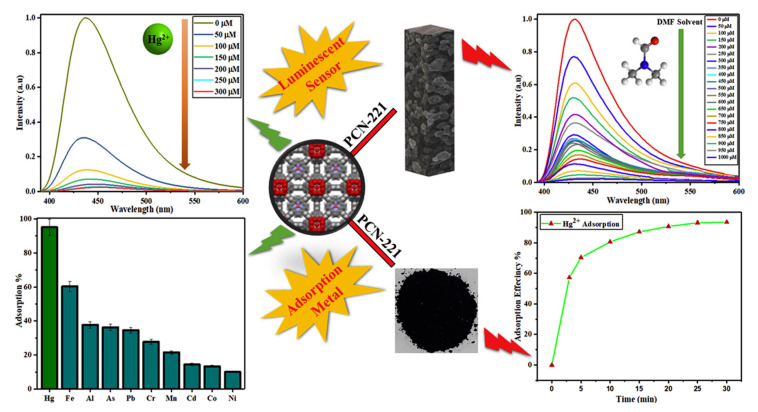
Porphyrinic zirconium-based MOF as an efficient fluorescence sensing for Hg^2+^ ions and DMF small molecule. Reprinted with permission from ref [62]. Copyright 2020 Elsevier Inc. All rights reserved.

Porphyrins and related macrocycles can act as attractive bridging ligands in assembling MOFs owing to their rigid molecular structures, tunable peripheral substituents, large physical dimensions, and an additional metalation site in the core. In addition, the well-isolated nature of porphyrin moieties can effectively weaken self-aggregation and enable their fluorescence response to the analytes. Compared with hierarchically porous materials, in which confined porphyrins are present inside their pores, porphyrin-based MOFs [111] have stable skeletons and abundant accessible recognition sites. Li et al. [56] have synthesized a porphyrinic metal–organic framework (MOF-525) as a fluorescent probe to target the Cu^2+^ ion with an LOD of 67 nM. Most of the reported porphyrin MOFs are copper ion detectors, such as Zr–MOF composite materials [encapsulating UiO-66(OH)_2_ into the porphyrin] [57] and MOF-525 nanoparticles (NPs) [58]. MOF-525 NPs with attractive properties, including ultrasmall size, good water dispersity, and intense red fluorescence, were prepared by the hydrothermal route, which is shown in Figure 12. The fluorescence signal of MOF-525 NPs can be statically quenched by Cu^2+^ with high selectivity owing to the strong affinity of Cu^2+^ to the porphyrin ligand in MOF-525. Xu et al. [59] have employed H_2_TCPP as a ligand and selected highly stable Zr_6_ clusters as nodes for the assembly of stable Zr-MOFs. They have synthesized MOF PCN-222 (Figure 13) and built a Chitosan–PCN222/GC sensor through the electrochemical method, utilizing the interaction between Cu^2+^ and the porphyrin core in PCN-222 to realize the sensitive detection of Cu^2+^ ions. PCN-224 exhibited a visible fluorescent quenching (bright red−dark red) and colorimetric response (purple−light green) in the presence of Hg^2+^ [61]. In 2020, Moradi et al. [54] have synthesized PCN-224 under solvothermal conditions by employing 5,10,15,20-tetrakis (4 carboxyphenyl) porphyrin (TCPP) as a ligand. At an excitation wavelength of 260 nm and an emission wavelength of 450 nm, PCN-224 exhibited fluorescence enhancement toward Cd^2+^. Moreover, PCN-224 could perform as a fluorescence “turn-off” sensor for the highly sensitive detection of THF in methanol solution. Recently, they have used TCPP as a ligand and synthesized a microporous zirconium-based MOF (PCN-221) [62]. As shown in Figure 14, this MOF can achieve the detection for Hg^2+^ and small molecules of dimethylformamide (DMF). With the porosity of MOF material, the PCN-221 can achieve high adsorption efficiency for Hg^2+^ ions. This is mainly due to the interaction of Hg^2+^ ions with a pyrrole Lewis base at the TCCP site. 

**Figure 15:**
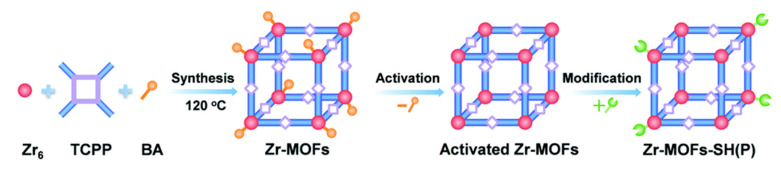
A schematic illustration of the synthesis of mercapto-functionalized Zr–MOFs (metal–organic frameworks) via the post-synthetic modification. Reprinted with permission from ref [112]. Copyright The Royal Society of Chemistry 2019.

**Figure 16:**
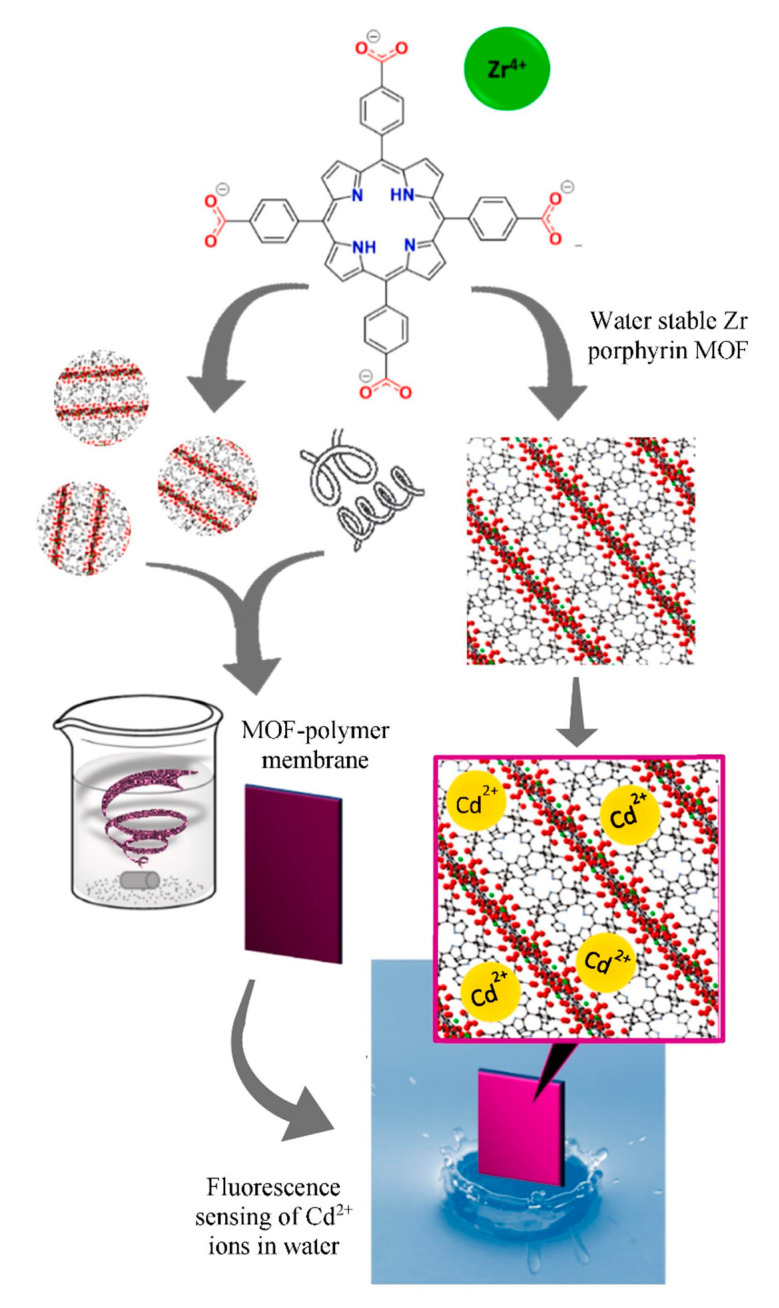
Schematic diagram of the detection for cadmium ions with MOF powder and polyvinyl chloride (PVC) membrane composites in aqueous solution. Reprinted with permission from ref [55]. Copyright 2020 Elsevier B.V. All rights reserved.

Since MOF has the characteristics of easy functional modification, Lin et al. [112] have used mercaptoacetic acid (MAA) or alpha lipoic acid (ALA) to synthesize mercapto-functionalized Zr-MOFs [Zr-MOFs-SH(O)], which had a much great adsorption capacity for Hg^2+^. The schematic illustration of the synthesis of mercapto-functionalized Zr-MOFs via the post-synthetic modification is shown in Figure 15. Chen and Jiang [60] have used a substitution reaction and non-fluorescent aniline to convert the Heck cross-coupling reaction on Pd NPs into a fluorescent closed-loop product. Using this change in fluorescence intensity, they developed a “turn-on” sensor for the Cu^2+^ detection. The LOD based on PCN-222-Pd (II) for Cu^2+^ was able to reach 50 nM. These porphyrin MOFs can be used to detect some metal ions because the interaction between porphyrins and metal ions can be detected. Considering the advantages and characteristics of different materials, a variety of materials can be combined to synthesize hybrid materials with dual functional characteristics. Wang et al. [63] have used the catalytic properties of gold nanoparticles and Fe–TCPP–MOF nano-simulated enzymes to combine the two materials to synthesize Au NP@MOF materials. On the basis of Hg^2+^ ion triggering Au catalytic methylene blue (MB) reduction, they established a colorimetric method with high sensitivity and the selective detection of Hg^2+^ ions. Hibbard et al. [55] have used a water-stabilized zirconium porphyrin-based metal–organic framework (NU-902) powder and fixed it in plasticized polyvinyl chloride (PVC) to form a film that was easy to use as a sensor in the field. The fluorescence of the MOF–PVC composite material was quenched after being exposed to an aqueous solution of Cd^2+^ for 5 min. The schematic diagram of the detection for Cd^2+^ ions in MOF powder and PVC membrane composites in aqueous solution is shown in Figure 16.

### 3.4. Graphene Materials

Graphene is composed of a single atomic layer of sp^2^-hybridized carbon atoms in the form of a two-dimensional hexagonal lattice and presenting a delocalized π cloud [113]. Each carbon atom contributes a π electron, and the electron can move freely in the graphene crystal [114]. Therefore, graphene has excellent conductivity and excellent mechanical, electrical, and optical properties, and it can be widely used in fields such as energy [115], biosensing [116], and electronic devices [117]. Thus, graphene hybrid materials were utilized to functionalize the porphyrins to show better detection performance for metal ions.

**Figure 17:**
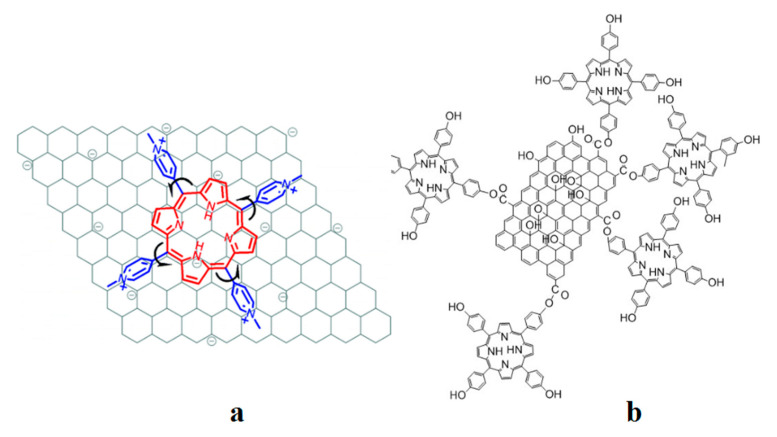
Molecular flattening of 5,10,15,20-tetrakis (4-*N*-methylpyridinyl) porphyrin *p*-toluenesulfonate (TMPyP) adsorbed on the chemically converted graphene (CCG) sheet (**a**) and the schematic illustration of the prepared graphene oxide–tetrakis (4-hydroxyphenyl) porphyrin (Go–THPP) (**b**). Reprinted with permission from ref [64,66]. Copyright 2009 American Chemical Society and The Royal Society of Chemistry 2015.

**Figure 18:**
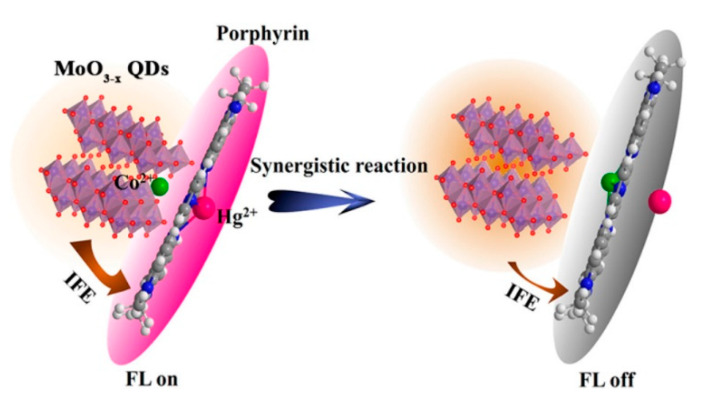
Strategy for Hg^2+^ sensing on the basis of the synergistic effect of MoO_3−x_ quantum dots (QDs) and Hg^2+^ on the cobalt porphyrin formation. Reprinted with permission from ref [68]. Copyright 2018 American Chemical Society.

As a type of well-known functional molecule, porphyrins can covalently functionalize graphene hybrid materials to make the materials have good optical limiting performance. In 2009, Xu et al. [64] have combined the diluted aqueous solutions of cationic TMPyP and negatively charged chemically converted graphene (CCG) sheets, which is shown in Figure 17. The electrostatic and π–π stacking synergistic interaction between the CCG sheet and TMPyP is the main driving force for complexation and molecular flattening. The flattening of TMPyP greatly accelerates the coordination reaction between Cd^2+^ ions and TMPyP, which achieves the rapid cadmium ion detection. Dorabei et al. [66] have synthesized graphene oxide–tetrakis (4-hydroxyphenyl) porphyrin (GO–THPP) to realize the detection of Hg^2+^ in water samples, which is shown in Figure 17, and the optimal pH was approximately 7.5. Different from the development of sensors by the direct use of graphene materials doped with porphyrins, functionalized graphene materials can be also used to catalyze the coordination reaction between metal ions and porphyrins, and the detection of metal ions can be realized using fluorescence changes. Zhang et al. [65] have used the coordination reaction between cadmium and TMPyP, which could be accelerated by nitrogen-doped graphene quantum dots (NGQDs). The coordination complex of Cd(NGQDs) can be rapidly associated with free TMPyP to form a molecular complex of Cd(NGQDs)·TMPyP, which labilizes coordinated water molecules and accelerates the incorporation of Cd^2+^ to form Cd(TMPyP). Moreover, the assembly of NGQDs and TMPyP via π−π stacking and electrostatic interaction facilitates the formation of the Cd(NGQDs)·TMPyP complex, which favors the incorporation of Cd^2+^ into TMPyP. Porphyrin combined with NGQDs can be used as a new optical probe for sensing Cd^2+^ ions. A team of researchers has also developed a method using nitrogen-doped graphene quantum dots to catalyze the formation of manganese porphyrin [67], and the combination of fluorescent molybdenum oxide (MoO_3−x_ QDs) with mercury ions was used to promote the formation of cobalt porphyrin [68] to achieve the detection of Hg^2+^. As shown in Figure 18, the synergistic reaction results in the rapid fluorescence/absorption spectral change of porphyrins upon metalloporphyrin formation as well as in the distinct fluorescence spectral evolution of MoO_3−x_ QDs owing to the inner filter effect (IFE) of porphyrins on MoO_3−x_ QDs, which realize the detection of metal ions. 

### 3.5. Other Materials

With the development of materials science, researchers have begun to design and use various functional materials to manufacture sensors with excellent performance. In addition to some of the above-mentioned materials, porphyrins can be assembled on MoS_2_ nanosheets to form nanocomposites, which can be used as optical sensors to detect Cd^2+^ ions in aqueous solutions [69]. Porphyrins can be attached to Au@SiO_2_ core/shell nanoparticles through covalent bonds and exhibit pronounced red fluorescence [74]. The porphyrin-functionalized Au@SiO_2_ core nanoparticles are recyclable sensors for fluorescent and colorimetric mercury. With its good biocompatibility and chemical stability, mesoporous silica has hydroxyl groups on its surface and can be combined with silane derivatives and inorganic substances. Most importantly, the mesopores in silica provide a certain space for loading the sensing unit and enriching the analyte. Tao et al. [80] have used a silane-derived porphyrin-modified mesoporous silica surface and the mesoporous, which was constructed by an amphiphilic quinolone molecule, and prepared a hybrid silica material with dual emission. This material could detect Al^3+^, Fe^3+^, Cr^3+^, and Fe^3+^, which can enhance the fluorescence of quinolone, while inhibiting the fluorescence of porphyrin. Cellulose nanocrystals doped with porphyrins [75], polyphosphazene porphyrin hybrid nanoparticles [76], upconversion nanoparticles [70], and mesoporous SBA-16 nanoparticles [79] can all be used as fluorescent nanoprobes to detect Hg^2+^.

The development of paper-based sensors relies on the use of colorimetry to develop a simple and fast detection method. Paper-based sensors rely on the use of cellulose paper as a platform to fix reporter molecules. With the help of imaging systems of micro-devices, such as mobile phones, color changes can be identified more finely. Since porphyrin is a colored compound, the colorimetric detection of metal ions can be achieved through cooperation with metal ions [71,72,73].

The sol–gel matrix has various advantages over other organic matrices, such as photochemical stability, variability in preparation conditions, hydrophilicity, and porosity of the resulting layer. Therefore, porphyrins can be fixed in a porous sol–gel to make a sensor element for metal ions detection, e.g., a sol–gel membrane sensor doped with water-soluble porphyrin, a hybrid supramolecular hydrogel mercury ion sensor containing Pd-porphyrin and gelling agents, and a tetrakis (4-pyridyl) porphyrin (TPyP) functionalized thermosensitive ion microgel (TPyP5-MGs) sensor [77,78,96].

When porphyrins are immobilized in other materials, other immobilized sensors with porphyrin properties can be fabricated. For example, porphyrin-based composite membranes can be used not only as electrochemical sensing membranes but also as optical sensor sensitive membranes. When porphyrin is doped into other nanomaterials, the constructed sensor not only has the optical properties of the single porphyrin molecule sensor, but also the unique adsorbability, conductivity, and catalytic properties of the material itself.

## 4. Conclusions and Perspectives

This review mainly introduces the detection of metal ions using porphyrin-based compounds. In addition, this review discusses this process in terms of the porphyrin fluorescent molecular sensor, porphyrin composite membrane sensor, and porphyrin composite nanomaterial sensor. In addition, we summarized the photochemical sensors developed for metal ions detection that are based on porphyrins. As shown in Table 1, there are 51 different types of optical sensors used to detect silver, copper, lead, cadmium, mercury, and iron, and most of them were utilized to detect heavy metals (e.g., cadmium, copper, lead, and mercury), because heavy metal ions can cause irreversible harm to the human body and the environment. It is worth mentioning that porphyrins have large extinction coefficients and fluorescence characteristics and are very attractive platforms for the development of chemical sensors. The rich chemical nature of these molecules offers many possibilities for ion detection. However, in terms of ion-specific detection, sensors developed by porphyrins still need to be improved. For example, (1) the detection environment of most sensors is in organic solvents; (2) when porphyrin is converted, the yield of the product is not high and does not meet the requirements of green development [30,33,40,81]; and (3) the types and number of porphyrin composite material sensors are limited. Therefore, it is possible to functionally modify the porphyrin outer ring substituent for adjusting its ion selectivity, and optimize the synthesis method of porphyrins for obtaining high-yield water-soluble porphyrin products in organic synthesis. In addition, the interaction between porphyrin and analyte can be handled by a variety of transducer mechanisms, which results in sensors with different characteristics. Therefore, suitable materials can be tailored for each specific application on the basis of their diversity and mechanistic knowledge. Moreover, with an increase in the demand for fast and convenient detection methods in modern society, and under the inspiration of Prabphal et al. [71], people can design more real-time, fast, and convenient porphyrin sensors by combining with electronic technology and mobile devices.

## Figures and Tables

**Table 1:** Summarized porphyrins and porphyrin-based materials for metal ions detection.

Type	Materials	Characteristics	Metal Ions	LOD	Reference
Molecular porphyrin	BODIPY-porphyrin dyad	Proportional detection of Ag^+^ in aqueous solution and living cells	Ag^+^	2.0 × 10^−7^ M	Zhu et al. [30]
	Porphyrin derivative	Neutral pH, low cytotoxicity, completed within 5 min, reversible	Cd^2+^	3.2 × 10^−8^ M	Huang et al. [31]
	THMPP	Highly photostable and water soluble	Cd^2+^	1.5 × 10^−8^ M.	Namitha et al. [32]
	Zinc porphyrin-dipyridylamino	Detection of Cu^2+^ in methanol solution, reversible	Cu^2+^	3.3 × 10^−7^ M	Weng et al. [33]
	Bis-TMPipEOPP	With a higher tendency to self-aggregate	Cu^2+^	8.8 × 10^−9^ M	Liu et al. [34]
	Porphyrin-quinoline dyad	In ethanol solution, pH 5.0–9.0, Hg^2+^ concentration in the range of 3 × 10^−7^–2 × 10^−5^ M	Hg^2+^	2.2 × 10^−8^ M	Han et al. [35]
	Thiourea derivative	Visualized under ultraviolet light, with membrane permeability and low toxicity, application to Hg^2+^ paper strips	Hg^2+^	1.8 × 10^−8^ M	Lv et al. [36]
	Tetrathia porphyrin	Colorimetric detection of Hg^2+^ in aqueous solution, high sensitivity	Hg^2+^	0.04 ppb	Virk et al. [37]
	Naphthalimide-porphyrin hybrid	Hg^2+^ concentration in the range of 1.0 × 10^−7^–5.0 × 10^−5^ M, reversible and fast (response time less than 2 min)	Hg^2+^	2.0 × 10^−8^ M	Li et al. [38]
	H_2_TEHPPS	High sensitivity and selectivity towards Mo^6+^ at pH 3.5	Mo^6+^	1.5 μg L^−1^	Feng et al. [39]
	H_2_Pc-α-ZnPor H_2_Pc-β-ZnPor	Pb^2+^concentration in the range of 0−4.0 μM, with dual-channel detection	Pb^2+^	3.4 × 10^−9^ M 2.2 × 10^−8^ M	Zhang et al. [40]
	H_2_Pc-β-(ZnPor)_2_	With a good linear relationship to Pb^2+^ concentration in the range of 0–2.0 μM	Pb^2+^	0.86 ppb	Qi et al. [41]
	Azacrown [N,S,O]-modified porphyrin	Simultaneous detection of Ag^+^, Pb^2+^ and Cu^2+^, Cu^2+^ with dual-mode detecting potential	Ag^+^	7.8 × 10^−7^ M	Chen and Wang [42]
			Cu^2+^	7.6 × 10^–13^ M	
			Pb^2+^	1.3 × 10^–11^ M	
	Cationic porphyrin	Ultraviolet detection of Hg^2+^, Pb^2+^, Cd^2+^, and Cu^2+^ in neutral aqueous solution	Cd^2+^ Cu^2+^ Hg^2+^ Pb^2+^	5 × 10^−7^ M 1.0 × 10^−6^ M 5 × 10^−7^ M 5 × 10^−7^ M	Zamadar et al. [43]
	ZnP-CONH-Q	With strong fluorescence enhancement	Y^3+^	-	Okamoto and Fukuzumi [44]
Porphyrin film	PAN/TMPyP/(PAH-PSS)_3_ membrane	Online visual and spectrophotometric sensing for cadmium during a flow-through process as well as static detection	Cd^2+^	1 ppm	Zhao et al. [45]
	PSF-PNaSS/TMPyP film	Visual and spectrophotometric detection and adsorption to remove Cd^2+^ ions, adjustable adsorption capacity, sensitivity, response time, and detection limit, the sensor membrane has good stability and reusability	Cd^2+^	−	Zhao et al. [46]
	TPPS/PVC membrane	Under alkaline conditions, the detection of Cd^2+^ within 20 min, and reversible	Cd^2+^	1.9 × 10^–5^ M	Czolk et al. [47]
	H_2_TPPBPy/PVC membrane	Cu^2+^ concentration in the range of 2.0 × 10^–8^−1.0 × 10^–5^ M, pH 6−8, reversible and fast (response time less than 5 min)	Cu^2+^	5 × 10^−9^ M	Luo et al. [48]
	DTPP/PVC membrane	Hg^2+^concentration in the range of 5.2 × 10^−7^−3.1 × 10^−4^ M, pH 2.4−8.0	Hg^2+^	5.2 × 10^−7^ M	Zhang et al. [49]
	Amphiphilic porphyrin film	Water-soluble and renewable	Hg^2+^	1 × 10^−5^ M	Dolci et al. [50]
	Self-assembled film	Hg^2+^ concentration in the range of 1 × 10^–10^–1 × 10^–6^ M and with high stability	Hg^2+^	1 × 10^–10^ M	Fang and Liu [51]
Metal complex	Ce_2_(TPP)_3_	Detection of Hg^2+^ and Cu^2+^ ions in aqueous solution with color change	Cu^2+^ Hg^2+^	2.34 × 10^−6^ M 1.6 × 10^−10^ M	Boroujerdi [52]
	Gd-TCPP	Colorimetric and fluorescent dual-mode detection of Fe^3+^, good water solubility, and determination of Fe^3+^ in fetal bovine serum samples	Fe^3+^	9.8 × 10^−8^ M	Chen et al. [53]
MOFs	PCN-224	Detection of Cd^2+^, Br^−^, and THF within 1 min	Cd^2+^	2 × 10^–9^ M	Moradi et al. [54]
	MOF-PVC composite	With fast and sensitive detection of Cd^2+^ ions	Cd^2+^	0.3 ppb	Hibbard et al. [55]
	MOF-525	High sensitivity in the Cu^2+^ ion concentration range of 0.1–1.2 mg L^−1^	Cu^2+^	6.7 × 10^−8^ M	Li et al. [56]
	UiO-66(OH)_2_@PCN-224	With built-in correction effect	Cu^2+^	6.8 × 10^−11^ M	Chen et al. [57]
	MOF-525 NPs	Linear range of 1.0−250 nM, detection of Cu^2+^ ion in water samples and living cells	Cu^2+^	2.2 × 10^–10^ M	Cheng et al. [58]
	PCN-222	The linear range of Cu^2+^ concentration is 0.4–13 μM, and the response time is less than 3 seconds	Cu^2+^	5.0 × 10^–8^ M	Xu et al. [59]
	PCN-222-Pd(II)	Fluorescence enhanced, detection of Cu^2+^ in complex environments	Cu^2+^	5.0 × 10^–8^ M	Chen and Jiang [60]
	PCN-224	Response rate as rapid as 2 min	Hg^2+^	6.0 × 10^–9^ M	Yang et al. [61]
	PCN-221	A quenching response of Hg^2+^ ions with a fast fluorescent response rate under <1 min.	Hg^2+^	1.0 × 10^–8^ M	Moradi et al. [62]
	AuNP@MOF	With fast response time, high sensitivity and selectivity	Hg^2+^	1.03 × 10^–10^ M	Wang et al. [63]
Graphene materials	TMPyP/CCG	Water-soluble	Cd^2+^	2 × 10^−6^ M	Xu et al. [64]
	TMPyP /NGQDs	Detection of Cd^2+^ within 2 min at 25 °C, pH 7.0	Cd^2+^	8.8 × 10^−8^ M	Zhang et al. [65]
	GO–THPP	Hg^2+^ concentration in the range of 6.0 × 10^−9^−6.0 × 10^−5^ M, pH 7.5, detection within 210 s, and with renewable ability	Hg^2+^	3.2 × 10^−9^ M	Dorabei et al. [66]
	TMPyP/NGQDs	Detection of Hg^2 +^ in pH 7.0 and phosphate buffer	Hg^2+^	3.2 × 10^−10^ M	Peng et al. [67]
	TMPyP/MoO_3−x_ QDs	Shorting analysis time, high sensitivity, intracellular imaging	Hg^2+^	8 × 10^−10^ M	Zhang et al. [68]
Other materials	MoS_2_@TMPyP	Molybdenum disulfide accelerating the formation of metal porphyrin	Cd^2+^	7.2 × 10^−8^ M	Yin et al. [69]
	UCNPs/TPPS	Using smartphones with color scanning APP to identify color changes in the detection process	Cu^2+^	1.3 × 10^−6^ M	Yan et al. [70]
	Paper-immobilized TMPyP	Combined with handheld UV lamp and smartphone or compact camera	Cu^2+^	0.16 ppm	Prabphal et al. [71]
	TSPP immobilized on paper	Detection within 5 min at neutral pH	Cu^2+^	1 × 10^−4^ M	Prabpal et al. [72]
	TDMPzP/Microfluidic paper	Suitable for testing under acidic conditions, pH 2.0−4.0	Cu^2+^	1 ppm	Pratiwi et al. [73]
	Porphyrin-functionalized Au@SiO_2_	Colorimetric fluorescence detection of Hg^2+^, color change within 10 s, with renewable fluorescence intensity	Hg^2+^	1.2 ppb	Cho et al. [74]
	CNC-SA-COOC_6_TPP	Good dispersion of cellulose nanocrystals	Hg^2+^	5 × 10^−8^ M	Chen et al. [75]
	TPP−PZS	With a color change in acetone solution and as a test strip for rapid detection of Hg^2+^	Hg^2+^	10 ppb	Hu et al. [76]
	Pd-TCPP/Supramolecular hydrogels	Hybrid gel bundle, small size, Hg^2+^ concentration in the range of 6 × 10^–8^−1 × 10^–6^ M	Hg^2+^	1.7 × 10^–11^ M	Xue et al. [77]
	TPyP5-MGs	Uniform distribution(radius about 189 nm), heat sensitive	Pb^2+^	5.9 × 10^−9^ M	Wen et al. [78]
	SBA-16@Porphyrin	Colorimetric and fluorescence detection of Hg^2+^, Pb^2+^ and Cu^2+^	Cu^2+^ Hg^2+^ Pb^2+^	3.6 ppm 3.8 ppm 5.8 ppm	Marcelo et al. [79]
	Amphiphile/porphyrin modified mesoporous silica	Dual-emission material	Al^3+^ Cr^3+^ Fe^3+^	2 × 10^−4^ M 1 × 10^−4^ M 8 × 10^−5^ M	Tao et al. [80]

## Abbreviations

8-HQ-B	8-Hydroxy Quinoline Benzoate
AIE	Aggregation-Induced Luminescence
ATRP	Atom Transfer Radical Polymerization
Au	Gold
Bis-TMPipEOPP	Bis-porphyrin (4,4’-bis[tris[4-[2-(1-methyl-1piperidinyl)ethoxy]phenyl] porphyrin
BODIPY	Boron–Dipyrromethene
CCG	Chemically Converted Graphene
CNCs	Cellulose Nanocrystals
CNC-SA-COOC6TPP	Synthesized by facile esterification of extended porphyrin with carboxylated cellulose nanocrystals
CP	Coordination Polymer
CMPSF	Chloromethylated Polysulfone
DMF	Dimethylformamide
DPA	2,2’-dipyridylamine
DTPP	*5-p*-[[4-(10’,-15’,20’-triphenyl-5’-porphinato)phenyloxyl]-1-butyloxyl]-phenyl-10,15,20-triphenylporphine
EDTA	Ethylenediaminetetraacetic acid
ESIPT	Excited Intra-molecular proton transfer
FRET	Fluorescence Resonance Energy Transfer
GO	Graphene Oxide
HPLC	High-Performance Liquid Chromatography
H_2_TEHPPS	5,10,15,20-tetra (3-ethoxy-4-hydroxy-5-sulfonate) phenylporphyrin
H_2_TPPBPy	A porphyrin derivative appended with bipyridine
IC	Ion Chromatography
ICP-MS	Inductively Coupled Plasma-Mass Spectroscopy
ICT	Intra-molecular Charge Transfer
IFE	Inner Filter Effect
LOD	Limit of Detection
MB	Methylene Blue
MOF-525	Porphyrinic MOF Zr_6_O_4_(OH)_4_(TCPP-H_2_)_3_
MOFs	Metal–Organic Frameworks
MoS_2_	Molybdenum Disulfide
NGQDs	Nitrogen-doped Graphene Quantum Dots
NPs	Nanoparticles
PAH	Poly(allylamine hydrochloride)
PAN	Polyacrylonitrile
Pc	Phthalocyanine
PCN-221	The microporous zirconium-based MOF
PCN-222	Porous coordination network is constructed using 5,10,15,20-tetrakis (4-carboxyphenyl) porphyrin (H_2_TCPP) as a heme-like ligand and highly stable Zr6 clusters as nodes
PCN-224	The coordination of Zr(IV) metal ions with meso-tetra(4-carboxyphenyl) porphyrin (TCPP) ligands produce MOF PCN-224); PET (Photo-induced Electron Transfer
PNaSS/PSS	Poly (sodium 4-styrenesulfonate)
Por	Porphyrin
PSF	Polysulfone
PSF-PNaSS	PNaSS-grafted polysulfone
PVC	Polyvinyl Chloride
QDs	Quantum Dots
SA	Succinic Anhydride
SBA-16	A mesoporous silica synthesized using tetraethoxysilane as a silicon source and a ternary combination of surfactant, water, and butanol
TCPP	5,10,15,20-tetrakis (4-carboxyphenyl) porphyrin
TDMPzP	Meso-tetrakis (1,2-dimethylpyrazolium-4-yl) porphyrin sulfonate
THPP	5,10,15,20-tetrakis (4-hydroxyphenyl) porphyrin
THMPP	5,10,15,20-tetrakis (4-hydroxy-3,5-dimethoxyphenyl) porphyrin
TMPP	5,10,15,20-tetrakis (3,4,5-trimethoxyphenyl) porphyrin
TMPyP	5,10,15,20-tetrakis (4-*N*-methylpyridinyl) porphyrin
TPP	Tetraphenylporphyrin
TPPS	5,10,15,20-tetrakis (*p*-sulfonatophenyl) porphyrin
TPP−PZS	Highly cross-linked poly(tetraphenylporphyrin-*co*-cyclotriphosphazene)
TPyP	Tetrakis (4-pyridyl) porphyrin
TPyP5-MGs	Tetrakis (4-pyridyl) porphyrin (TPyP) functionalized thermosensitive ion microgel
UCNPs	Upconversion Nanoparticles
UiO-66(OH)_2_	A MOF named by University of Oslo
UV	Ultra-Violet
ZnP-CONH-Q	Zinc Porphyrin-Quinone-Linked Dyad
Zr-MOFs-SH(O)	Mercapto-functionalized Zr-MOFs
